# Towards Identifying Protective B-Cell Epitopes: The PspA Story

**DOI:** 10.3389/fmicb.2017.00742

**Published:** 2017-05-02

**Authors:** Naeem Khan, Arif T. Jan

**Affiliations:** ^1^Glycobiology Group, Max Planck Institute of Colloids and Interfaces (MPG)Potsdam, Germany; ^2^Department of Medical Biotechnology, Yeungnam UniversityGyeongsan, South Korea

**Keywords:** antibodies, epitopes, hybridoma, pneumococcal surface protein A, *Streptococcus pneomoniae*

## Abstract

Pneumococcal surface protein A (PspA) is one of the most abundant cell surface protein of *Streptococcus pneumoniae* (*S. pneumoniae*). PspA variants are structurally and serologically diverse and help evade complement-mediated phagocytosis of *S. pneumoniae*, which is essential for its survival in the host. PspA is currently been screened for employment in the generation of more effective (serotype independent) vaccine to overcome the limitations of polysaccharide based vaccines, providing serotype specific immune responses. The cross-protection eliciting regions of PspA localize to the α-helical and proline rich regions. Recent data indicate significant variation in the ability of antibodies induced against the recombinant PspA variants to recognize distinct *S. pneumoniae* strains. Hence, screening for the identification of the topographical repertoire of B-cell epitopes that elicit cross-protective immune response seems essential in the engineering of a superior PspA-based vaccine. Herein, we revisit epitope identification in PspA and the utility of hybridoma technology in directing the identification of protective epitope regions of PspA that can be used in vaccine research.

## Introduction

*Streptococcus pneumoniae* (or pneumococcus) is a Gram-positive bacterium that asymptomatically colonizes the upper respiratory tract of humans. Colonization of the nasopharynx by *S. pneumoniae* involves adherence of the bacterium to the epithelial surface via different surface molecules (McCullers and Tuomanen, 2001). On invading the host immune system, *S. pneumoniae* can migrate to the lungs (pneumonia), blood (bacteremia), middle ear (otitis media) and sometime cross the blood-brain barrier (meningitis) in humans (Boulnois, 1992; Mook-Kanamori et al., 2011). Pneumococcal diseases can cause high mortality in children, the elderly and immunocompromised patients. With more than 90 distinct serotypes, the transition from asymptomatic nasopharyngeal carriage of *S. pneumoniae* to invasive pneumococcal disease depends on the balance between the host’s defense mechanisms and bacterial adherence ability, nutrition and their replication within the host (Bridy-Pappas et al., 2005). Of the available vaccines, 23-valent capsular polysaccharide vaccine (23-PPV) is ineffective in children less than 2 years of age (Barocchi et al., 2007), while as 7-valent glyconjugate vaccine (7-PCV) is effective but has limited serotype coverage (Cremers et al., 2015). Lately two vaccines, 10-valent and 13-valent glyconjugate vaccines has been licensed for use in humans, while as 15-valent vaccine is currently under consideration (Prymula and Schuerman, 2009; Bryant et al., 2010). Given serious consideration to limited serotype coverage, there is utmost need to have serotype independent vaccine; generated solely on the protein based strategy or using proteins as candidate in conjugate vaccines, for making them effective against broader range of *S. pneumoniae* serotypes.

Pneumococcal surface protein A (PspA) is one of most abundant surface molecules and a major determinant of protective immunity. Study of the role of PspA in virulence through insertion duplication mutagenesis revealed that PspA is essential for nasopharynx colonization (McDaniel et al., 1987). Addition to its role in lung infection and bacteremia (Ogunniyi et al., 2007), PspA prevents phagocytosis by inhibiting complement-mediated opsonization of the bacterial cells (Ren et al., 2004). With high genetic variability, this choline binding protein with molecular size ranging from 67 to 99 kDa, is employed for analyzing the global distribution of pneumococci (Crain et al., 1990; Hollingshead et al., 2006). On one side where serotype diversity of *S. pneumoniae* complicates the generation of effective vaccines, use of proteins seems advantageous to overcome the limitation with the existing vaccines. To this, PspA is a promising vaccine candidate because genomes of all *S. pneumoniae* isolates harbor the *pspA* gene.

## Structural Analysis of PspA

Though PspA was originally identified by protective monoclonal antibodies (mAbs) raised in CBA/N mice (McDaniel et al., 1984, 1986), cloning of full length PspA gene helped in predicting the complete amino acid sequence of PspA (Yother and Briles, 1992). Based on C-terminus α-helical domain, PspA is categorized into three cross-reacting families with >55% identity and six clades, with >75% identity, of which clades 1 and 2 are included in family 1, clades 3, 4, and 5 in family 2, and clade 6 in family 3, respectively (Hollingshead et al., 2000; Khan et al., 2015). Most of the pneumococcal isolates belonging to PspA family 1 and family 2 (Beall et al., 2000; Brandileone et al., 2004; Hotomi et al., 2013). With so much diversity between clades, it becomes imperative to have an understanding of different structural aspects of PspA, to confine regions that offer serotype independent protection against varied *S. pneumoniae* serotypes.

On analyzing the N-terminal half of PspA from the clade 2 strain Rx1 against known members of other clades, sequence homology of amino acids was found in the range of 45 (EF3296) -78% (BG9739) (Jedrzejas et al., 2001). As α-helical part of the protein is capable of tolerating vast number changes in the amino acid sequence, PspA sequences across different serological groups are found to have a central coiled-coil part flanked by different structural domains (Briles et al., 1998). Despite sharing less identity in the α-helical residues, PspA molecules are structurally conserved in terms of the position of hydrophobic residues that contribute more to the maintenance of coiled structure in the α-helical region (Yother and Briles, 1992). As such, conserved residual position of hydrophobic residues rather than dissimilarity of coiled structure residues appears a contributing factor to the biological property of PspA.

**FIGURE 1:**
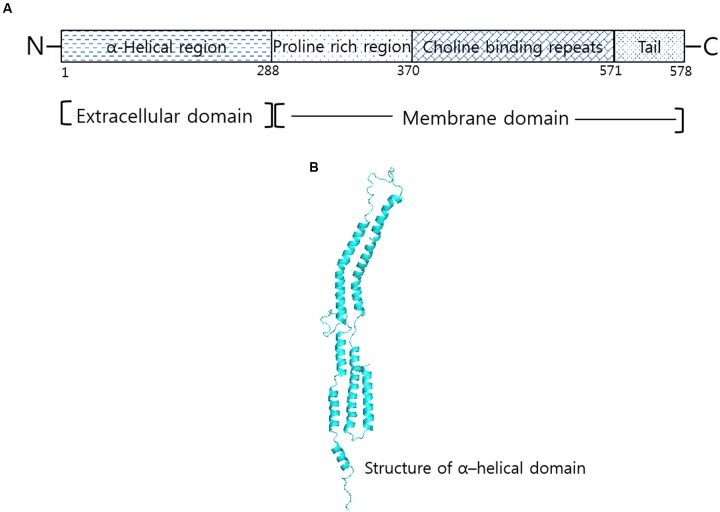
**Structural correlation of PspA in *Streptococcus pneumoniae*. (A)** Domain organization of PspA in R36A strain show α-helical region, proline rich and choline binding repeat regions. **(B)** Structure corresponding to α-helical domain of PspA generated by Swiss modeler online server (https://swissmodel.expasy.org/) and validated using Ramachandran plot of Rampage server tool (http://mordred.bioc.cam.ac.uk/~rapper/rampage).

Having four domain structural arrangements (**Figure 1A**), analysis of N-terminal half (1–288 amino acid residues) of PspA from strain Rx1 show a seven-residue periodicity in non-polar amino acid distribution (Yother and White, 1994). Compared to N-terminal part that shows higher presence of net negative charge, the C-terminus of PspA contains a proline rich region (289–371), 10 repeats of 20 conserved amino acids that depict a choline binding domain and a hydrophobic region of 17 amino acid residues. Despite sharing antiphagocytic activity, difference in the primary structure of PspA was found among the heterologous serotypes studied (Tu et al., 1999). Furthermore, the appearance of a fibrous network on the cell surface suggests resemblance of PspA to previously characterized tropomyosin and M proteins. However, sequence check of PspA with tropomyosin and streptococcal M proteins revealed only 45% amino acid similarity in the seven-residue periodicity of non-polar amino acids of the α-helical region of PspA (McLachlan and Stewart, 1975; Hollingshead et al., 1986; Fischetti, 1989). Different from the heptad repeat of streptococcal M-protein that triggers autoimmune response, there are no reports that suggest cross-reactivity of PspA to human proteins (Briles et al., 2000). Representing safe for use in humans, this advantageous property of PspA is exploited for use as vaccine candidate; for which it is currently undergoing clinical trials.

Structural conformation prediction study firmly confirms resemblance of PspA to fibrous proteins (Yother and Briles, 1992). Of the seven-residue periodicity (a-b-c-d-e-f-g)_n_ repeat, the hydrophobic residues (a, c) were found associated with the coiled core formation and the remaining (b, d, e, f, and g) was found having role in promoting the helix formation. Similarity in structure corresponding to 1 through 288 amino acid residues suggests a central coiled-coil conformation of PspA from the strain Rx1 (Jedrzejas et al., 2000). Discontinuity in the heptad sequence from other PspA and coiled-coil molecules generally account for the flexibility of PspA as observed in the electron micrographs (Hollingshead et al., 1986; Jedrzejas et al., 2001). The flexible nature of PspA arises on account of the presence or absence of disruptions in the periodicity of hydrophobic residues. Differing across the PspA molecules and even among the same serotype strains, the predominance of helix-promoting residues in the heptad allows PspA molecules to attain similarity among α-helical conformations (**Figure 1B**). Existence of PspA as a monomer through ultracentrifugation and spectra observed in the circular dichroism study indicates PspA of having more than 70% α-helical content (Jedrzejas et al., 2000). Studies on the crystal structure of lactoferrin and the PspA provides evidence for PspA existing as an α-helical coiled- coil structure (Senkovich et al., 2007).

## Mechanistic Characterization of PspA

### Complement Inhibition

**FIGURE 2:**
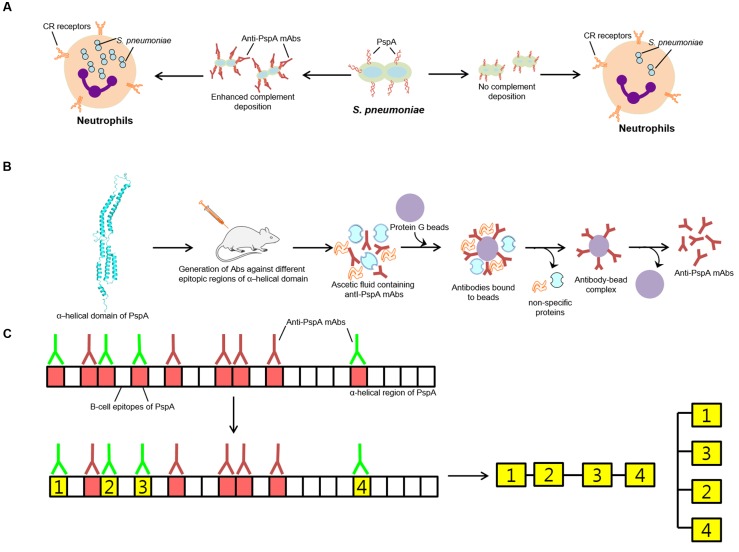
**Role of PspA in protection. (A)** PspA imparting resistance against engulfment by neutrophils. Pneumococcus showing more PspA on the surface shows less complement deposition and as such less killing by neutrophils. **(B)** Mechanism adapted for the generation of anti-PspA- mAbs. **(C)** Graphical demonstration of the production and characterization of antibodies (anti- PspA-mAbs) generated against different combinations of selected PspA epitopes of Red and green color antibodies represent non-protective and protective antibodies, respectively. The green block represents epitopic regions of PspA that elicit generation of protective antibodies.

As an important component of the immune system, deposition of the complement components on the surface of pneumococcus is facilitated by the absence of PspA or by the addition of anti-PspA antibodies (**Figure 2A**), which results in the faster clearance of pneumococci by phagocytic cells bearing the C3b receptors (Carroll, 1998; Ren et al., 2012). Thus, pneumococci lacking the pspA gene get cleared faster by the immune system compared to wild type pneumococci (Joiner et al., 1983; McDaniel et al., 1987; Briles et al., 1998). Amount of C3 deposited on the surface of pneumococcus being higher in PspA^-^ compared to PspA^+^ strains, suggest enhancement in the clearance of pneumococci from the body (Ren et al., 2004, 2012). Consistent with the notion that PspA^+^ pneumococci resist phagocytosis, evidences of faster opsonization via alternative pathway of PspA^-^ pneumococci is reported (Tu et al., 1999; Cheng et al., 2000; Brown et al., 2002; Yuste et al., 2006). Toward mechanistic elucidation, Mukerji et al. (2012) showed that the surface bound PspA inhibited C3 deposition by competing with the C-reactive protein. However, higher variability of PspA (classified into three family and six clades) highlights the importance of having specific knowledge of the epitopic regions associated with complement mediated phagocytosis.

### Lactoferrin Binding

As a member of the transferrin family of iron-binding glycoproteins, lactoferrin is reported to be expressed by glandular epithelial cells and secreted into the tissue secretions like colostrums, milk, saliva, nasal wash, and tears (Ward et al., 2005). It is also produced by neutrophils as secondary granules (Masson et al., 1969; Berliner et al., 1995). Hammerschmidt et al. (1999) demonstrated that PpsA interact with the iron saturated lactoferrin. The lactoferrin binding property of PspA was found exclusive for the human lactoferrin presumably because humans are the only known natural hosts for *S. pneumoniae* (Hakansson et al., 2001). The lactoferrin binding property has little to do with the vaccine design, as it constitutes another important property of bacteria that helps it to adhere with the surface of the host. The co-crystal structure of N-terminal domain of lactoferrin with PspA revealed that lactoferrin interact strongly with the helix 3 and helix 4 (Senkovich et al., 2007). However, it is not clear that how pneumococci utilize the human lactoferrin in pathogenesis. Previous reports have shown that anti-PspA antibodies enhance the killing of pneumococci by apolactoferrin (Shaper et al., 2004; Bitsaktsis et al., 2012; Mirza et al., 2016).

## Role of PspA in Virulence and Immunogenicity

Pneumococcal surface protein A attributed virulence to the *S. pneumoniae* is essential for nasopharynx colonization, and in causing lung infection and bacteremia (Ogunniyi et al., 2007). PspA elicits a high level of antibodies in humans, as antibodies to PspA were found in the sera of infected individuals (Briles et al., 2000; Melin et al., 2008, 2012). The protective ability of PspA was analyzed when mice were given PspA^-^ and PspA^+^ unencapsulated strain Rx1 and challenged with the strain WU2 (McDaniel et al., 1987). Active immunization with PspA in animal models was found to confer protection against the nasopharyngeal carriage and invasive disease (Wu et al., 1997). Mice immunized with DNA vaccine expressing the N-terminal region of PspA were found protected against the intraperitoneal challenge with a strain expressing heterologous PspA (Ferreira et al., 2006). Daniels et al. (2010) demonstrated that the proline-rich region of PspA contains surface-accessible epitopes that are protective in both active and passive mouse protection experiments.

Irrespective of the serological variability, PspA expression is observed in all clinically relevant capsular serotypes (Crain et al., 1990). Immunization of mice with recombinant PspA elicited antibodies produced in humans passively protects it upon infection with pneumococci (Briles et al., 2000). Furthermore, regions of diverse PspA variants homologous to the 192–588 amino acid region of strain Rx1 were found highly immunogenic and as such cross-protective against unrelated strains of pneumococci (Tart et al., 1996). The rabbit antisera raised against the recombinant PspA from strain Rx1 (clade 2 PspA), exhibited cross reactivity with all six clades of PspA (Nabors et al., 2000). Similarly, Darrieux et al. (2008) analyzed recognition of a panel of 35 pneumococcal isolates bearing diverse PspA variants by antisera raised against the N-terminal region of PspA from clade 1 to clade 5. The antisera against the PspA of clades 4 and 5 were found cross-reactive against pneumococcal strains expressing PspAs of all clades from families 1 and 2. The cross-reactivity of antibodies elicited against a PspA hybrid which included the N-terminal region of clade 1 fused to C-terminal α-helical domain (∼100 amino acid residues) of PspA from clade 4, exhibited strong binding with pneumococcal lysates of clades 1, 4, and 5 and a weak binding with lysate from clade 2 to clade 3 (Darrieux et al., 2007). The polyclonal sera against PspA/Rx1 provided significant cross protection against challenge with the virulent strains when passively transferred into the mice (Briles et al., 2000). The active immunization of PspA from family 2 containing α-helical domain and proline-rich region provided significant cross protection when challenged with the strains from PspA families 1 and 2, while immunization with the α-helical domain of family 2 PspA alone was found involved in providing family specific protection (Moreno et al., 2010; Kothari et al., 2015). Using monoclonal antibodies, Kolberg et al. (2001) found that individual mAb cross-reacted with 20–50% strains and in combination could recognize about 94% of the strains analyzed (Kolberg et al., 2001). With higher cross reactivity, antibodies generated against PspA held greater possibility of providing cross-protection against varied pneumococcal strains (Tart et al., 1996; Briles et al., 2000; Andre et al., 2015; Kristian et al., 2016). Despite sequence divergence, cross-reactivity of PspA observed in most of the studies on PspA makes it a potentially promising vaccine candidate.

## Epitope Mapping Using anti-PspA Monoclonal Antibodies

Attributing *S. pneumoniae* with the anti-phagocytic property, use of recombinant PspA leads to production of protective antibodies similar to capsular polysaccharide. With evidences suggesting that all anti-PspA antibodies are not protective, it becomes imperative to have an understanding of the epitopes that can elicit protective antibody response. Epitope mapping is currently been employed in the identification of cross protecting antibodies generated against different epitopic regions of PspA to access their contribution in providing protection across different clades of PspA, for employment in the generation of superior serotype independent vaccine. To localize protection eliciting regions of PspA, McDaniel et al. (1994) raised a series of nine mAbs against PspA from strain Rx1. Of them, five mAbs attributing protective behavior on infecting mice with a virulent strain were mapped to N-terminal 115 amino acids and 192–260 amino acid stretches of PspA from the strain Rx1. Compared with mAb that correspond to N-terminal 115 amino acids residues, four mAbs recognizing 192–260 amino acid regions were found more cross-reactive (**Figures 2B,C**).

Recognizing different subset of pneumococcal strains, seven anti-PspA mAbs were divided into two groups on the basis of their reactivity. It was found that all epitopes recognized by these mAbs were surface accessible (Kolberg et al., 2001). Surprisingly, a combination of the two anti-PspA mAbs detected 94% of the 77 strains analyzed. In a study, Darrieux et al. (2008) reported anti-PspA antibodies generated against family 2 PspAs, which showed more cross reactivity across different clades compared with those generated against family 1 PspAs that showed limited cross-reactivity within the family. Rohatgi et al. (2009) reported generation of anti-PspA mAbs against surface exposed domain of PspA from R36 strain. In their study, they suggested that these antibodies exhibit diverse VH and Vk genes/families. Furthermore, characterization of anti-PspA mAbs revealed that seven mAbs that encoded DH-less heavy chain gene did not affect to attain the average relative avidity. Compared with the recombinant PspA, immunization of mice with heat-killed R36A resulted in generation of anti-PspA polyclonal antibodies that too with higher avidity (Rohatgi et al., 2009). In a similar type of studies, mAbs generated against proline-rich region (PPR) of PspA from Rx1 strain were found more cross- reactive (Daniels et al., 2010; Melin et al., 2012). These studies clearly indicated the potential benefit of identifying protective B-cell epitopes of PspA that show conserved nature across all PspA clades.

## Medical Perspective

Emergence of non-vaccine serotypes poses a great challenge in the management of pneumococcal diseases. These concerns are driving efforts to develop a ‘universal’ pneumococcal vaccine that is immunogenic in all age groups and broadly cross protective against all serotypes. Efforts are being made to develop a serotype independent protein based vaccine to prevent pneumococcal infections. Rather than targeting a single candidate protein, targeting a complex of proteins based on their roles in bacterial pathogenicity and physiology seems appropriate. Several studies have reported use of two or more proteins for achieving additive and broad protection against pneumococci in mice (Briles et al., 2003; Ogunniyi et al., 2007; Chen et al., 2015; Lagousi et al., 2015).

The rapid emergence of resistance to antimicrobials like penicillin has complicated the global management of pneumococcal disease. The polysaccharide vaccines have several shortcomings which include its limited serotype coverage and poor immunogenicity in high-risk groups. To this, replacement of the vaccine serotypes by other non-vaccine serotypes is currently being perused. However, increasing the number of serotypes in the vaccine increases the cost of preparation that may limit its deployment in under-developing and developing countries. These concerns are driving efforts to develop universal pneumococcal vaccine that is immunogenic in all age groups and broadly cross protective against all serotypes. As proteins are antigenically conserved across epidemiologically relevant serotypes, it is assumed that coupling proteins (**Figure 2C**) with potential to act as effective immunogens in a multi-component protein-based pneumococcal vaccine or as a carrier protein in conjugate vaccine would confer broader resistance to pneumococci. With this, protein based vaccines are considered as a better replacement of capsular-polysaccharide based vaccines.

Study of the prevalence of seven different protein candidates including PspA within global (445 isolates from 26 countries representing four continents) serotype 1 collection of *S. pneumoniae*, revealed only 68% (305/445) coverage for possible implementation as a vaccine candidate (Cornick et al., 2017). Individual distribution of PspA among serotype 1 study population showed presence of PspA among 76% of Asian isolates, 67% of African isolates, 68% of European isolates and 41% among South American isolates. Though, monovalent usage showing limited coverage, multivalent vaccine candidate combinations (PspA combination with CbpA, PcpA, and PhtD) increased global serotype coverage of PspA from 68 to 86%. Taking a note of this, multivalent vaccine having PspA as a component advocates increased serotype coverage relative to monovalent vaccine candidates. Increases in the efficaciousness of protein based vaccines in combinations thereby offers an effective vaccine intervention to the disease across the globe, irrespective of the type and geographical distribution of *S. pneumoniae*.

Represented as hotspot of recombination events, PspA undergoes highest number of transforming events to evade host antibody response (Croucher et al., 2017). To this, identification of conserved protection eliciting B-cell epitopes of PspA holds great promise in engineering a superior PspA-based vaccine. Knowledge of protective epitopes can also be employed in the generation of fusion construct of multi-epitopes or used for developing a covalent conjugate with well-defined dendrimers or cyclodextran for direct employment as a multiantigenic semi-synthetic immunogen (Gupta et al., 2012). Additionally, these epitopes can also be used as an important constituent in the preparation of conjugate vaccines with the pneumococcal polysaccharide. Collectively, PspA seems an appropriate option to be selected as one of the candidates that can be employed to reduce the global burden of pneumococcal diseases.

## Author Contributions

NK conceived the idea; NK and AJ equally contributed to writing of the manuscript and to preparation of figures.

## Conflict of Interest Statement

The authors declare that the research was conducted in the absence of any commercial or financial relationships that could be construed as a potential conflict of interest.
